# Prediction of mortality and morbidity following paraquat poisoning based on trend of liver and kidney injury

**DOI:** 10.1186/s40360-022-00609-y

**Published:** 2022-09-06

**Authors:** Farzad Gheshlaghi, Jamileh Haghirzavareh, Anselm Wong, Parastoo Golshiri, Shayan Gheshlaghi, Nastaran Eizadi-Mood

**Affiliations:** 1 Department of Clinical Toxicology, School of Medicine; Isfahan Clinical Toxicology Research Center, Isfahan University of Medical Sciences, Isfahan, Iran; 2 Medical Practitioner, Department of Clinical Toxicology, School of Medicine, Isfahan University of Medical Sciences, Isfahan, Iran; 3 Victorian Poisons Information Centre, Austin Toxicology and Emergency Department Austin Health, Heidelberg, and Department of Medicine, School of Clinical Sciences at Monash Health, Monash University, Clayton, VIC Australia; 4 Department of Community Medicine and Family Physician, Isfahan University of Medical Sciences, Isfahan, Iran; 5 Medical Practitioner, School of Medicine, Islamic Azad University Najafabad Branch, Isfahan, Iran

**Keywords:** Paraquat, Liver injury, Kidney injury, Poisoning

## Abstract

**Background:**

Paraquat is a non-selective herbicide that causes severe tissue damage in various organs including the liver and kidney. The aim of this study was to determine the trend of the liver and kidney injury in patients with paraquat poisoning.

**Methods:**

This retrospective cross-sectional study was performed at the Khorshid Hospital referral poisoning emergency center. The medical records of all patients with acute paraquat poisoning admitted from March 2017 to October 2020 were reviewed. Demographic factors, liver and kidney function tests and outcomes were recorded. Patients were divided into two groups based on the outcome of mortality (death or survived). The two groups were compared in terms of changes in creatinine and liver enzymes during hospitalization.

**Results:**

A significant difference in mean creatinine levels between the two groups was observed from the third day after admission. The peak median Cr was 3.5 mg/dl for deceased patients in day 6 and 1.47 mg/dl for survived patients on 4th day. Minor elevations of ALT and AST were present in those who died. Logistic regression analysis shows patients who had level of creatinine higher than normal from the 2nd to 6th day post overdose, the risk of mortality was 4.83 to 7.44 times more than patients with normal creatinine level. The mean (SD) area under the curve for outcome prediction was reported to be excellent for creatinine on the 8th day post overdose (85.7 ± 13.2). Creatinine was higher than 2 on the 8th day post ingestion and had a sensitivity 100% and specificity 85.7% for mortality prediction (*P* value, 0.05).

**Conclusions:**

The risk of mortality secondary to paraquat ingestion was highly associated with a rise in creatinine. Minor elevations of ALT and AST were also present in those who died. The creatinine concentration on different days post overdose can be helpful in predicting the severity of poisoning especially when the serum paraquat levels are not available.

## Background

Paraquat is a non-selective herbicide that is widely used in many developing countries [1]. Clinical manifestations of paraquat toxicity are mainly due to the production of intracellular reactive oxygen species, which cause cell damage through lipid peroxidation, activation of nuclear factor kappa B, mitochondrial damage, and apoptosis in many organs [1]. By the same mechanism, paraquat causes damage to the lungs, myocardium, kidneys, liver, adrenal glands, and central nervous system and can lead to multiple organ failure [2–4]. The liver is one of the organs that is affected primarily by the mechanism of damage to mitochondria and degeneration of the endoplasmic reticulum, and the severity of this liver damage is manifested by the speed and intensity of liver enzymes increase [5, 6]. The kidneys are another organ that is damaged in paraquat poisoning which leads to an increase in creatinine level by vacuolation of proximal tubular nephron cells and tubular necrosis [7, 8].

Despite the variety of treatments proposed for paraquat poisoning [9–11], the mortality rate is very high at about 50 to 90% [4]. There have been some studies regarding the association of liver and kidney injury with mortality. In a study by Suji Kim et al. (2009), serum creatinine levels above 1.2 μg/dl were associated with increased mortality [12]. In another study conducted by Yi-soo et al. (2020), serum creatinine was one of the predicting factors for clinical outcome in patients with paraquat poisoning [13].

Although there have been reports of the association of mortality with liver injury secondary to paraquat toxicity, these have not been conclusive. Acute liver failure was considered as a cause of mortality in paraquat poisoning in the study by Kanchan et al. [14] However, there was no difference in mortality rates between patients with and without hepatic complication in another study [15]. Therefore, we aimed to evaluate the trends of acute kidney and liver injury in patients with acute paraquat poisoning and its association with mortality.

## Methods

This is a cross-sectional study performed using data from the Khorshid Hospital toxicology referral center affiliated with the Isfahan University of Medical Sciences. All patients with acute paraquat poisoning who were admitted to the toxicology referral center from March 2017 to October 2020 were included in the study; therefore, the sampling method was census during the mentioned period.

The diagnosis of paraquat poisoning was based on a positive urinary dithionate test [16] and history of ingestion. Patients with a negative dithionate urine test during the first 24 hours of hospitalization, or who were discharged against medical advice, or had a history of chronic kidney and liver diseases were excluded from the study. All included patients received treatments based on local protocols [17–19].

Data were collected by reviewing the patient medical records. Information recorded in the data collection form included demographic variables (age, sex), onset of clinical signs, liver function tests (aspartate aminotransferase (AST) and alanine aminotransferase (ALT) in IU/L) and renal function (creatinine in μg/dl) during hospitalization. The primary outcome was in-hospital mortality. The collected data were analyzed using SPSS software version 16. Ethics approval for the study was given by the ethics committee of Isfahan University of Medical Sciences (project number IR.MUI.MED.REC.1399.612).

Categorical data were compared using Chi-square or Fisher’s exact tests. Proportions were compared using the two-way repeated measure ANOVA or independent T-test. Binary logistic regression analysis (backward conditional stepwise method) was used to calculate odds ratio (OR) as the estimate of the relative risk of the different parameters for the prediction of mortality. Discrimination was tested using the area under the Receiver Operating Characteristic (ROC) curves and 2 × 2 classification matrices [20] Area under the curve (AUC) between 0.7 and 0.8 were classified as ‘acceptable’ and between 0.8 and 0.9 as ‘excellent’ discrimination [21]. The area under the ROC curve, sensitivity, and specificity and the best cutoff point were determined. The best cut-off point was that which maximized the sum of specificity and sensitivity in the ROC analysis. This cutoff point was also used to calculate the predicted and observed mortality. The *P* value of less than 0.05 was considered as statistically significant.

## Results

During the study period, 64 patients were hospitalized due to paraquat poisoning. Of these, 23 patients were excluded because of a negative dithionate test within 24 hours. Forty-one (64.06%) patients met study criteria. The mean (SD) age of the patients was 28 (± 8.6) years. Most of the patients were male (*n* = 36, 87.8%). The mean (SD) creatinine (Cr), AST and ALT at the admission time were 1.0(± 0.4) μg/dl; 24.7(±18.1) IU/L; and 20.4 (±14.4) IU/L respectively. Eighteen patients (44%) died and all of them were male (*P*-value = 0.03) (Table 1).

**Table 1:** Demographics of the study population with respect to mortality

Parameters	Survived [22]	Died [18]	Total (41)	*P* value
Age (year), mean (SD)	27 ± 9	30 ± 7	28 ± 8	0.23
Gender, n (%)				
Men	18 (78.3)	18 (100)	36 (87.80)	0.03
Women	5 (21.7)	0 (0)	5 (12.20)	

The Fisher’s exact and independent T-test was used

**Fig. 1:**
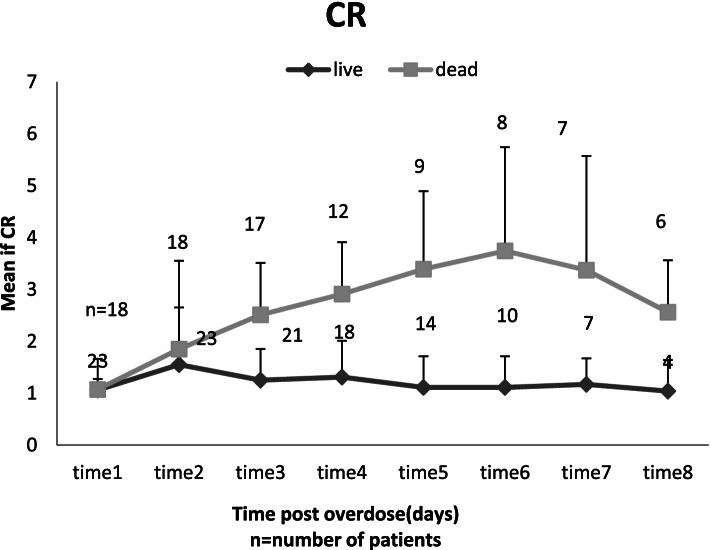
The trend of creatinine (CR) in paraquat poisoned patients during hospitalization with respect to mortality (survived/death)

The changes in blood creatinine of surviving patients did not show a significant difference during hospitalization (*p* = 0.26), but in the deceased patients, a significant upwards trend was observed (*p* < 0.001). There was no significant difference in demographics between the two groups by adjusting the effect of gender (*p* = 0.08). A significant difference in mean creatinine levels between the two groups was observed from the third day after admission. The maximum creatinine level was observed in the deceased patients on the sixth day of hospitalization (Fig. 1). There was also a significant difference in median Cr during admission between patients with respect to mortality (*P* < 0.05). The peak median Cr was 3.5 μg/dl for deceased patients on day 6 and 1.47 μg/dl for survived patients on 4th day.

**Fig. 2:**
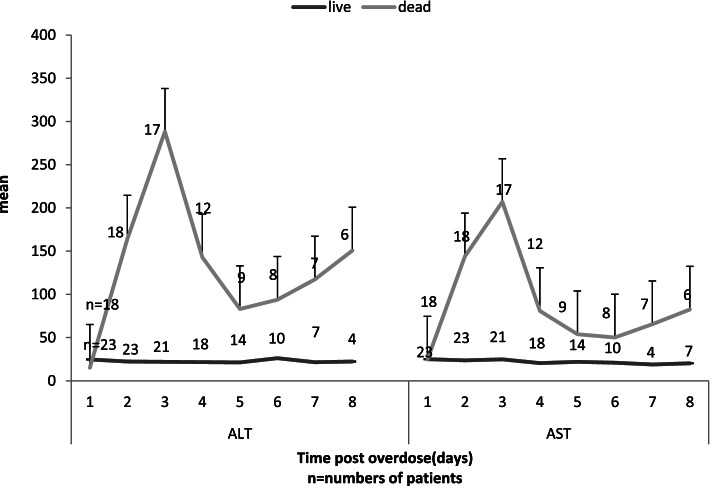
The trend of alanine transaminase (ALT) and aspartate transaminase (AST) in paraquat poisoning patients during hospitalization with respect to outcome (survived/death)

The mean AST in surviving patients were not significantly different during hospitalization days (*p* = 0.88), but there was a significant upwards trend in deceased patients (*p* = 0.04). There was no significant difference between the demographics of the two groups when adjusting for sex (*p* = 0.98). The mean AST from the second day showed a significant difference between those who survived and mortality groups (*P* < 0.001) (Fig. 2). There was no significant rise in ALT on admission in survived cases (*p* = 0.42). In contrast a substantial peak was recorded on 3th day (*P* < 0.001) in those who died (Fig. 2).

Binary regression analysis showed that a rise in creatinine which peaked in those who survived at 6th day was greatly linked with mortality. However, the ALT and AST had a minimal increase in those who died. This was not associated with an increased risk of mortality (Table 2). The area under the curve of the variables studied was excellent in the first day for creatinine. (Table 3).

**Table 2:** Prediction of mortality based on the level of creatinine (Cr), alanine transaminase (ALT) and aspartate transaminase (AST) during hospitalization in patients with paraquat poisoning

^a^Variable	Unadjusted			^η^Adjusted		
	β	*P* value	OR (95% CI)	β	*P* value	OR^a^ (95% CI)
Cr (3th day)	1.93	0.003	6.91 (1.96–24.35)	2.01	0.001	7.47 (2.17–25.72)
Cr (4th day)	2.22	0.002	7.44 (2.04–27.04)	2.00	0.002	7.44 (2.04–27.04)
Cr (5th day)	1.79	0.009	6.02 (1.57–23.16)	1.79	0.009	6.02 (1.56–23.16)
Cr (6th day)	1.57	0.047	4.83 (1.02–22.843	1.57	0.047	4.83 (1.02–22.84)
AST(5th day)	0.05	0.051	1.05 (1.00–1.11)	0.05	0.051	1.05 (1.00–1.11)
ALT(3th day)	0.02	0.042	1.02 (1.00–1.05)	0.02	0.030	1.03 (1.00–1.05)
ALT(4^th^day)	0.04	0.050	1.04 (1.00–1.10)	0.04	0.050	1.04 (1.00–1.10)

^a^numbers in the variable shows the day of enzyme measurement; η, adjusted for the age and gender

**Table 3:** Prediction of mortality based on the level of creatinine (Cr), alanine transaminase (ALT) and aspartate transaminase (AST) and Areas under the ROC curves during hospitalization for mortality

Component	AUC *±* SE (95% CI)	Cutoff point	Specificity	Sensitivity	*P* value
Cr (2th day)	77.4 ± 7.9	0.99	94.4	56.5	0.003
Cr (3th day)	87.3 ± 6.5	1.20	94.1	66.7	0.000
Cr (4th day)	88.2 ± 7.9	1.96	91.7	83.3	0.000
Cr (5th day)	87.7 ± 10.2	1.67	88.9	85.7	0.003
Cr (6th day)	85.0 ± 11.8	2.10	87.5	90.0	0.01
Cr (7th day)	81.6 ± 13.5	2.15	71.4	100	0.04
Cr (8th day)	85.7 ± 13.2	2.00	85.7	100	0.05
AST (3th day)	92.3 ± 4.3	25.50	100	52.4	0.000
AST (4^th^day)	85.1 ± 9.4	26.00	81.1	83.3	0.002
AST(7^th^day)	88.8 ± 9.6	25.50	85.7	85.7	0.01
AST (8^th^day)	87.5 ± 12	30.50	85.7	100	0.04
AST(9^th^day)	100	21.50	100	100	0.04
ALT(3th day)	82.8 ± 7.2	20.50	76.5	71.4	0.001
ALT(4^th^day)	82.2 ± 9.1	24.00	83.3	72.2	0.003
ALT(5^th^day)	77 ± 10.6	26.00	66.7	85.7	0.03
ALT(7^th^day)	83.7 ± 13.3	23.50	85.7	85.7	0.03
ALT(8^th^day)	89.3 ± 10.8	34.50	85.7	100	0.03

## Discussion

The aim of this study was descriptive on serial LFTs and renal function which has not plotted in detail versus time previously. The results of our study showed that most of the patients with paraquat poisoning were male and this was similar to other studies [22, 23]. All patients who died were also male. Most patients were young and there was no significant difference between the mean ages of patients with respect to outcome which was similar to other studies show [16, 17, 20–25].

Patients who ingested paraquat and survived were unlikely to have a rise in creatinine concentrations compared to those who died. The levels of creatinine on the 3th to 8th days after ingestion were highly predictive of mortality. A study conducted by Suji Kim et al. showed that a serum creatinine level higher than 2.1 was associated with increased mortality [12]. They also reported that those who have a rise in creatinine within 24 hours are at high risk of mortality [12]. In our study, the highest sensitivity (100%) and specificity (85%) to predict mortality related to the rise in creatinine occurred on the eighth day post ingestion of paraquat, with the cutoff level of 2 μg/dl. The logistic regression model also showed that patients who had level of creatinine higher than normal from the 2nd day to 6th day post ingestion of paraquat had an increased risk of mortality (4.83 to 7.44 times more than patients with normal creatinine level). Results show that liver enzymes became mildly elevated in the patients that died on the second and third days post ingestion. However, liver injury was not associated with an increased risk of mortality. Although the risk of mortality secondary to paraquat ingestion was highly associated with a rise in creatinine, Cr gradually increased and peaked at Day 6; whereas ALT and AST rapidly elevated and peaked at Day 3. Variability was large in AST and ALT, but it may be partly due to the small our study population. Therefore, we suggest a larger validation study to be performed to confirm our findings.

Multi-organ failure due to paraquat toxicity is a well-established feature of paraquat toxicity. Given there is no antidote, and mortality is very high, prognosis/prediction is important to help distinguish those who are at higher risk of mortality. It can prepare the patient and family for who has a high likelihood of dying. However, for the prediction of liver and kidney toxicity, a larger prospective compressive study with long term follow up assessment is suggested.

Also, due to the fact that serum paraquat levels were not available to determine the severity of toxicity in many poisoning treatment centers, a significant rise in creatinine or mild rise in liver enzymes may help predict mortality. This study is unique and specific to paraquat poisoning. There have not been other studies with serial creatinine and ALT levels with respect to outcome.

There are some limitations in our study. There may be missing history given the retrospective nature of the study, however, laboratory results and case history were obtainable from the case records. Also, this was a single site study and may not be generalizable to different settings. In addition, paraquat concentrations were not available to correlate degree of poisoning severity.

## Conclusions

The risk of mortality secondary to paraquat ingestion was highly associated with a rise in creatinine. Minor elevations of ALT and AST were also present in those who died. Although, there were no other causes of liver injury identified in this study and all presentations were related to ingestion of paraquat, our current study may not determine the association of mortality with liver injury definitively.

## Data Availability

The data that support the findings of this study are available from the authors.
